# *NTRK2* expression levels are reduced in laser captured pyramidal neurons from the anterior cingulate cortex in males with autism spectrum disorder

**DOI:** 10.1186/s13229-015-0023-2

**Published:** 2015-05-16

**Authors:** Michelle J Chandley, Jessica D Crawford, Attila Szebeni, Katalin Szebeni, Gregory A Ordway

**Affiliations:** Department of Health Sciences, College of Public Health, East Tennessee State University, P.O. Box 70673, Johnson City, TN 37614 USA; Department of Biomedical Sciences, James H. Quillen College of Medicine, East Tennessee State University, P.O. Box 70582, Johnson City, TN 37614 USA

**Keywords:** Pyramidal neurons, Astrocytes, Cingulate, Autism, Glutamate receptors

## Abstract

**Background:**

The anterior cingulate cortex (ACC) is a brain area involved in modulating behavior associated with social interaction, disruption of which is a core feature of autism spectrum disorder (ASD). Functional brain imaging studies demonstrate abnormalities of the ACC in ASD as compared to typically developing control patients. However, little is known regarding the cellular basis of these functional deficits in ASD. Pyramidal neurons in the ACC are excitatory glutamatergic neurons and key cellular mediators of the neural output of the ACC. This study was designed to investigate the potential role of ACC pyramidal neurons in ASD brain pathology.

**Methods:**

Postmortem ACC tissue from carefully matched ASD and typically developing control donors was obtained from two national brain collections. Pyramidal neurons and surrounding astrocytes were separately collected from layer III of the ACC by laser capture microdissection. Isolated RNA was subjected to reverse transcription and endpoint PCR to determine gene expression levels for 16 synaptic genes relevant to glutamatergic neurotransmission. Cells were also collected from the prefrontal cortex (Brodmann area 10) to examine those genes demonstrating differences in expression in the ACC comparing typically developing and ASD donors.

**Results:**

The level of *NTRK2* expression was robustly and significantly lower in pyramidal neurons from ASD donors as compared to typically developing donors. Levels of expression of *GRIN1*, *GRM8*, *SLC1A1*, and *GRIP1* were modestly lower in pyramidal neurons from ASD donors, but statistical significance for these latter genes did not survive correction for multiple comparisons. No significant expression differences of any genes were found in astrocytes laser captured from the same neocortical area. In addition, expression levels of *NTRK2* and other synaptic genes were normal in pyramidal neurons laser captured from the prefrontal cortex.

**Conclusions:**

These studies demonstrate a unique pathology of neocortical pyramidal neurons of the ACC in ASD. *NTRK2* encodes the tropomyosin receptor kinase B (TrkB), transmission through which neurotrophic factors modify differentiation, plasticity, and synaptic transmission. Reduced pyramidal neuron *NTRK2* expression in the ACC could thereby contribute to abnormal neuronal activity and disrupt social behavior mediated by this brain region.

**Electronic supplementary material:**

The online version of this article (doi:10.1186/s13229-015-0023-2) contains supplementary material, which is available to authorized users.

## Background

Autism spectrum disorder (ASD) is a neurodevelopmental disorder that includes repetitive behaviors and impairments in social communication and interaction [1]. In recent years, ASD and disorders associated with autism behaviors have largely been attributed to genetic etiology [2]. However, it is likely that the heterogeneity of ASD results from a complex interplay of inherited genetics and developmental influences that result in abnormal intercellular communication in the brain. It is anticipated that areas of the brain that modulate the behaviors that are disrupted in ASD are particularly vulnerable to the pathobiological processes of ASD and likely include disrupted connectivity between discrete areas of the brain that modulate behaviors that are abnormal from infancy.

The anterior cingulate cortex (ACC) is the cortical gyrus surrounding the corpus callosum in the human brain. Anatomically, the ACC shares a complex relationship with several other brain regions including the amygdala, hypothalamus, parietal, and other regions of the prefrontal cortex (as reviewed by Bush *et al*., 2000 [3]). The ACC has consistently displayed abnormalities in ASD as revealed through imaging and neuroanatomical studies. Functional MRI studies have demonstrated differences when comparing typically developed controls with ASD patients, including reduced activation of the anterior cingulate cortex (ACC) during true/false judgments [4], novelty detection [5], response inhibition [6,7], attention tasks [8], and increased activation of the ACC during social target detection and nonsocial rewarding [9]. Other studies using SPECT [10], EEG [11-14], PET [15-18], and fractional anisotropy [19,20] have also demonstrated abnormalities in the cingulate cortex in ASD.

The few postmortem studies that have focused on the ACC have identified molecular and anatomical differences between ASD and typically developing control brain tissue. Stereology studies using the neocortex of the ACC indicated that neurons were smaller and demonstrated increased cell packing density [21]. Reductions in both mRNA and protein levels of the axonal guidance proteins plexinA4 and roundabout 2 were identified in tissue homogenates from the ACC from ASD donors [22], as well as alterations in serotonin- [23] and GABA- [24,25] related genes. Interestingly, increased gene expression in the transcriptional control factor, Sp1, was found in postmortem ACC from ASD donors [26], which could have widespread implications and contribute to the complexity of ASD. Collectively, these studies demonstrate that pathology in the ACC exists in autism. However, little is known regarding the cellular basis of ACC pathology.

Pyramidal neurons in layer III of the neocortex are key cellular mediators of the neural output of the ACC. These pyramidal cells are excitatory glutamatergic neurons that have a complex synaptic relationship with many other cell types in other neocortical layers of the ACC, including inhibitory neurons, glia, and long-range projecting glutamate neurons of layer V. The current study was undertaken to investigate the molecular pathology of pyramidal cells in layer III of the ACC from ASD donors. The levels of expression of several glutamate-related genes were measured specifically in pyramidal neurons captured by laser microdissection from layer III of the ACC from postmortem brain tissue from ASD donor and typically developing control subject brain tissue. The glutamate-related genes chosen for study were those associated with ASD identified in gene association, laboratory animal, and/or postmortem pathology studies [27-32] and/or because of their strong association with glutamatergic neurotransmission. Additionally, two neurotrophic factor genes (brain-derived neurotrophic factor (*BDNF*) and *NTRK2*) were studied because of the link between glutamatergic transmission and BDNF signaling [33] and the association of BDNF and *NTRK2* pathology in ASD [34]. The findings of this study demonstrate that ACC in ASD is associated with abnormal levels of expression of several genes related to glutamate neurotransmission, with the most striking finding being a robust reduction of *NTRK2* gene expression.

## Methods

### Brain tissue

Brain tissues from ASD and typically developing control donors were provided by the National Institutes for Child Health and Development (NICHD) Brain and Tissue Bank (Baltimore, MD) and the Autism Tissue Program (Belmont, MA). These brain banks were responsible for obtaining subject consent and the unidentifiable coding of subject information. This study was reviewed and approved for exemption by the Institutional Review Board of East Tennessee State University under the Department of Heath and Human Services exemption 45 CFR 46.101(b) relating to the use of publicly available unidentifiable pathology specimens. In total, brain tissue from 12 typically developing control donors and 12 ASD donors were used for the different experiments (see Table 1). Comorbidities and causes of death were not included in the table in order to protect donor identities. Typically developing control donors died by drowning (3 donors), heart condition (3 donors), trauma (3 donors), asphyxia (1 donor), pneumonia (1 donor), and unknown cause (1 donor). It should be mentioned that one control donor was diagnosed with depressive disorders and died by suicide. The ASD donors died by trauma (3 donors), asphyxia (3 donors), acute respiratory distress syndrome (1 donor), cardiopulmonary arrest (1 donor), cancer (1 donor), diabetic ketoacidosis (1 donor), bowel obstruction (1 donor), and cardiac arrhythmia (1 donor). One ASD donor could not be medically confirmed as ASD after death, and one ASD donor had a single seizure episode but did not have a medical diagnosis of seizure disorder. The samples were closely matched by gender and age. For each control and ASD pair, the donor tissue came from the same brain bank and was not anatomically characterized by subregions of Brodmann area 24 (BA24) or Brodmann area 10 (BA10). Additionally, we analyzed RNA integrity values (RIN, index of RNA quality) in matched pairs of donors prior to experimentation to be sure these were closely matched for the paired analyses [35].

**Table 1:** **Subject demographic information**

**Pair**	**ID**	**Age**	**Gender**	**RIN** ^**a**^	**PMI (hours)** ^**b**^	**Toxicology**	**Assays**	**Tissue**
Control donors								
1	AN14757	24	M	7.8	21.33	No drugs reported	LCM^c^, qPCR^d^	BA24, BA10
2	AN07176	21	M	8	29.91	No drugs reported	LCM, qPCR	BA24, BA10
3	AN07444	17	M	7.5	30.75	Sertraline	LCM, qPCR	BA24
4	5408	6	M	7	16	No drugs reported	LCM, qPCR	BA24, BA10
5	4848	16	M	7.6	15	No drugs reported	LCM, qPCR	BA24, BA10
6	5342	22	M	8.1	14	No drugs reported	LCM, qPCR	BA24, BA10
7	5079	33	M	7.3	16	Ethanol	LCM, qPCR	BA24, BA10
8	M3231M	37	M	7.4	24	No drugs reported	LCM, qPCR	BA24, BA10
9	4590	20	M	7.6	19	No drugs reported	LCM, qPCR	BA10
Mean ± SEM (BA24 donors)		22.0±3.4		7.59±0.13	20.9±2.4			
Mean ± SEM (BA10 donors)		22.4±3.4		7.60±0.13	19.4±1.9			
ASD donors								
1	AN04166	24	M	8.1	18.51	No drugs reported	LCM, qPCR	BA24, BA10
2	AN03935	20	M	8.6	28	No drugs reported	LCM, qPCR	BA24, BA10
3	AN02987	15	M	6.5	30.83	No drugs reported	LCM, qPCR	BA24
4	5144	7	M	8.0	3	No drugs reported	LCM, qPCR	BA24, BA10
5	5302	16	M	6.6	20	Risperidone, fluvoxamine, clonidine, insulin	LCM, qPCR	BA24, BA10
6	5176	22	M	7.1	18	Risperidone	LCM, qPCR	BA24, BA10
7	5297	33	M	7.1	50	Quetiapine, fluoxetine, valproate, ziprasidone	LCM, qPCR	BA24, BA10
8	5027	37	M	7.7	26	Risperidone, fluvoxamine	LCM, qPCR	BA24, BA10
9	4999	20	M	7.0	14	No drugs reported	LCM, qPCR	BA10
Mean ± SEM (BA24 donors)		21.6±3.4		7.46±0.27	24.3±4.8			
Mean ± SEM (BA10 donors)		22.2±3.3		7.53±0.24	23.4±5.4			
P *value* ^*e*^ *(BA 24)*		0.94		0.68	0.53			
P *value* ^*e*^ *(BA10)*		0.98		0.79	0.60			

^a^RNA integrity number (index of RNA quality); ^b^postmortem interval; ^c^endpoint PCR analysis of reversed transcribed RNA isolated from cells collected by laser capture microdissection; ^d^quantitative polymerase chain reaction of reverse transcribed RNA isolated from punch-dissected tissues; ^e^results of a two-tailed independent *t*-test comparing control and ASD groups. BA24, Brodmann area 24; BA10, Brodmann area 10; LCM, laser capture microdissection.

### Tissue preparation

Blocks of tissue containing ACC, specifically BA24, and prefrontal cortex BA10 were received and stored at −80°C. All brain tissues were obtained from the left hemisphere except AN03935. Tissue homogenates used for quantitative PCR were obtained using a 3.5-mm trephine to punch-dissect a 50-μm-thick section of gray matter from the anterior cingulate that contained all six neocortical layers. A cryostat microtome was used to section tissue blocks for laser capture microdissection (LCM). Tissue from the cortex was sectioned at −20°C and 10-μm-thick sections were mounted slides that were immediately placed in a chilled microslide box on ice. Between each tissue block, all physical elements of cryosectioning were thoroughly cleaned with 100% ethanol to avoid any cross contamination. Sectioned tissue was desiccated at room temperature for 5 min and stored at −80°C until use. Tissues from the same control/ASD donor pair were sectioned and processed on the same day to ensure that storage time after sectioning was identical.

### Laser capture microdissection

**Figure 1:**
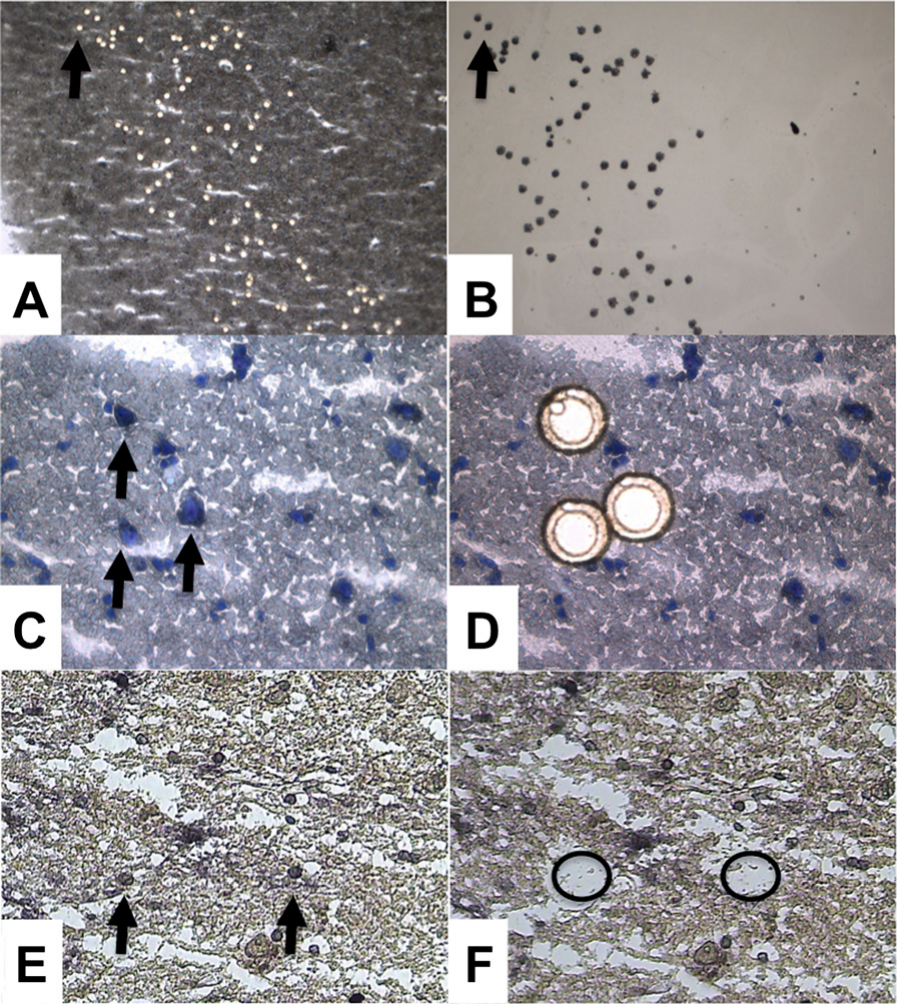
Laser capture microdissection of pyramidal neurons and astrocytes. Shown in **(A)** is the absence of laser captured nissl-stained pyramidal neurons from cortical layer 3 in BA24 gray matter tissue following capture (20× magnification). **(B)** shows those cells adhered to the polymer cap (20× magnification). **(C)** and **(D)** illustrate the before (C) and after (D) capture images for nissl-stained BA24 pyramidal neurons (40× magnification). **(E)** and **(F)** show the same laser capture process for GFAP immunostained astrocytes in BA24 gray matter (60× magnification).

Neurons were visualized by staining frozen tissue sections with the Histogene staining kit (Life Technologies; Grand Island, NY, USA) according to the manufacturer’s instructions (Figure 1C). In short, the protocol used a cresyl violet stain on an ethanol-fixed slide followed by a series of dehydration steps. Stained slides were placed in a vacuum chamber until ready for LCM. Astrocytes were identified using a modified glial fibrillary acidic protein (GFAP) rapid immunohistochemistry protocol as previously described (Figure 1E) [36,37]. Briefly, tissue-mounted slides were fixed in acetone (5 min), blocked in horse serum (10 min), incubated with GFAP antibody (10 min; ThermoFisher; Pittsburgh, PA, USA), anti-mouse antibody (5 min; Vectastain; Burlingame, CA, USA), and avidin-biotinylated horseradish peroxidase (5 min; Vectastain; Burlingame, CA, USA). GFAP immunoreactivity was visualized by exposure to diaminobenzidine (DAB) (Sigma; St. Louis, MO, USA) with ammonium nickel sulfate (ANS) (Sigma; St. Louis, MO, USA) for 5 min, then exposed to DAB/ANS with 0.1% H_2_O_2_ for an additional 5 min. Afterwards, the slide was ethanol dehydrated then placed in xylene for 5 min. The slide was kept in a vacuum chamber for 10 min prior to LCM.

LCM was performed using an ArcturusXT (Life Technologies; Grand Island, NY, USA) instrument. Neurons were extracted onto CapSure macrocaps (Life Technologies; Grand Island, NY, USA) using a 25-μm spot size that is first placed onto the cap by infrared (IR) laser spotting and then cut by an ultraviolet (UV) laser. Neurons were morphologically identified in layer III of the neocortex at 40× magnification. Astrocytes were captured using an Arcturus Veritas (Life Technologies; Grand Island, NY, USA) instrument. Astrocytes were morphologically identified at 60× magnification and placed onto caps by IR laser using a 10-μm spot size. Cells were removed from cap using lysis buffer incubation at 42°C.

### RNA preparation and reverse transcriptase

Total RNA was extracted from tissue homogenates using the Maxwell simplyRNA LEV kit (Promega; Madison, WI, USA) and from laser captured cells using the RNAqueous Micro kit with DNase treatment (Life Technologies; Grand Island, NY, USA). RNA quality was assessed by measuring RIN values obtained using the Agilent 2100 Bioanalyzer (Agilent Technologies; Santa Clara, CA, USA). RNA samples were reverse transcribed into cDNA using the Superscript III kit (Life Technologies; Grand Island, NY, USA) that contained oligodT and random hexamer primers.

### Quantitative PCR

Gene-specific primers were either designed using Mfold web server software [38] and primer quest design software (IDT; Coralville, IA, USA) to generate approximately 100 base pair amplicons to span exon junctions or were purchased from a vendor (Qiagen; Valencia, CA, USA). Gene primer sequences are shown in Additional file 1. To quantify transcripts, endpoint PCR was used for RNA isolated from laser captured cells and real-time quantitative PCR (qPCR) was used for RNA isolated from tissue homogenates as previously described [36,39]. For endpoint PCR reactions (*BDNF*, *GRIN2D*, *GRIN2B*, and *GRM8*) that were initially problematic using 5Prime Hot Master Mix taq polymerase (5Prime; Gaithersburg, MD, USA), a modified polymerase from Qiagen (Valencia, CA, USA) was employed using the same reaction parameters.

### Statistical analysis

Calculations for qPCR data involved converting cycle threshold (CT) values to fold-change between control and autism donors using the 2^−ΔΔCT^ method by Livak and Schmittgen [40]. Endpoint PCR data was computed as relative values generated from the ratios of amounts of target gene expression to the average of two reference gene expressions. Afterwards, both data from qPCR and endpoint PCR were analyzed by the paired Student *t*-test. Statistical results are reported before and after Holm’s Bonferroni correction [41,42] for the number of comparisons as noted in the results below. Pearson’s correlation was used to determine possible effects of postmortem variables (age, RIN, and postmortem interval time (PMI)) on the expression of each gene. Given the number of correlation tests, a *P* < 0.01 was chosen *a priori* to indicate statistical significance in order to reduce type I errors. Multivariate analysis of variance (MANOVA) for unpaired data was performed using IBM SPSS Statistics (version 21.0.0.0, IBM, New York, NY, USA) and graphed using Prism (version 5.0b, GraphPad Software, La Jolla, CA, USA). All other analyses were performed and graphed using Prism.

## Results

### Glutamate-related gene expression

**Figure 2:**
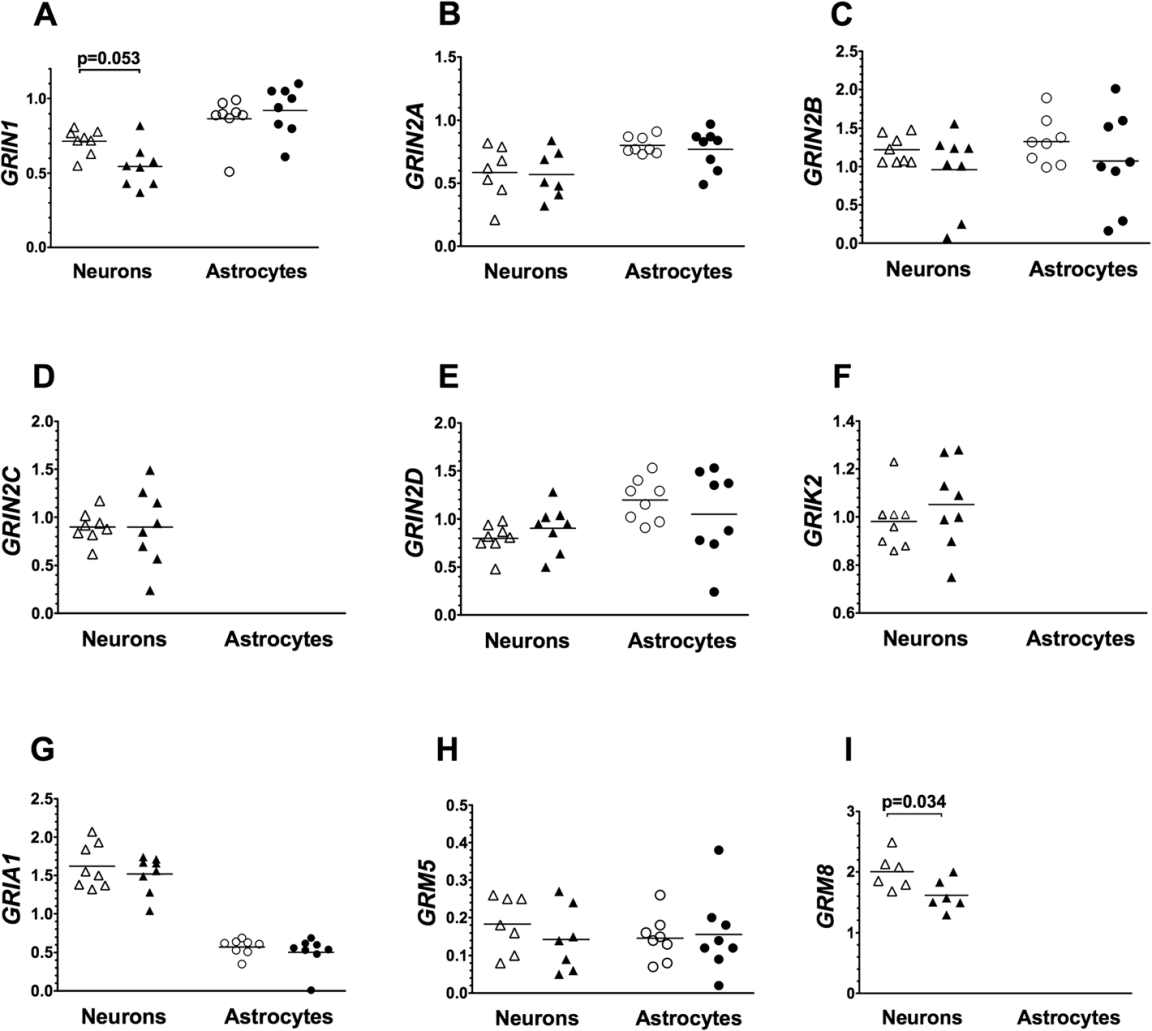
Levels of expression of ionotropic glutamate receptor subunits and metabotropic glutamate receptors **(A-I)**. Expression of glutamate receptors and receptor subunits was measured in laser captured BA24 pyramidal neurons and separately in surrounding astrocytes of typically developing control donors (open symbols) and ASD donors (closed symbols). Gene expression levels are normalized to the averaged levels of expression of references genes (*GAPDH* and *RNA18S1*). Mean values are noted by horizontal lines, and statistical significance (uncorrected for the number of comparisons) is noted above the data points. See Table 2 for statistical results following correction for the number of comparisons.

The levels of expression of seven ionotropic glutamate receptor subunit genes (*GRIN1*, *GRIN2A*, *GRIN2B*, *GRIN2C*, *GRIN2D*, *GRIK2*, *GRIA1*) and two metabotropic glutamate receptor genes (*GRM5*, *GRM8*) were measured in BA24 pyramidal neurons and surrounding astrocytes from ASD and age-matched control donors. *GRM8* expression was not detectable in astrocytes. Levels of *GRM8* expression were modestly lower in pyramidal neurons from ASD donors compared to matched control donors (*t* = 2.89; *P* = 0.034), but statistical significance was lost when the *P* value was corrected for the number of matched pair comparisons of gene expressions in neurons (Table 2). Likewise, *GRIN1* expression levels trended towards being lower in ASD as compared to control donors (*P* = 0.053), but this difference was lost upon correction for the number of comparisons (Table 2). No other differences in the levels of expression of any glutamate receptor gene were observed in either pyramidal neurons or astrocytes comparing ASD to control donors (Figure 2).

**Table 2:** **Results of Holm-Bonferroni sequential correction of multiple paired Student’s** ***t*** **-tests of gene expression data from BA24 neurons**

**Gene (protein)**	***P*** **value from paired** ***t*** **-test**	***P*** **’ from correction** ^**a**^	**Significance?**
*RNA18S1/GAPDH*	0.3774	1.000	No
*GRIN1* (NR1)	0.0533	0.583	No
*GRIN2A* (NR2A)	0.9051	1.000	No
*GRIN2B* (NR2B)	0.1160	1.000	No
*GRIN2C* (NR2C)	0.9924	1.000	No
*GRIN2D* (NR2D)	0.1146	1.000	No
*GRIA1* (GluR-1)	0.2424	1.000	No
*GRIK2* (GRIK2, GluK2)	0.3793	1.000	No
*GRM5* (mGluR5)	0.1061	1.000	No
*GRM8* (mGluR8)	0.0340	0.408	No
*SLC1A1* (EAAT3)	0.0235	0.312	No
*SLC17A7* (VGlut1)	0.5034	1.000	No
*GRIP1* (GRIP1)	0.0064	0.090	No
*BDNF* (BDNF)	0.4846	1.000	No
*NTRK2* (NTRK2,TrkB)	0.0006	0.009	Yes

^a^Holm-Bonferroni corrected *P* value.

**Figure 3:**
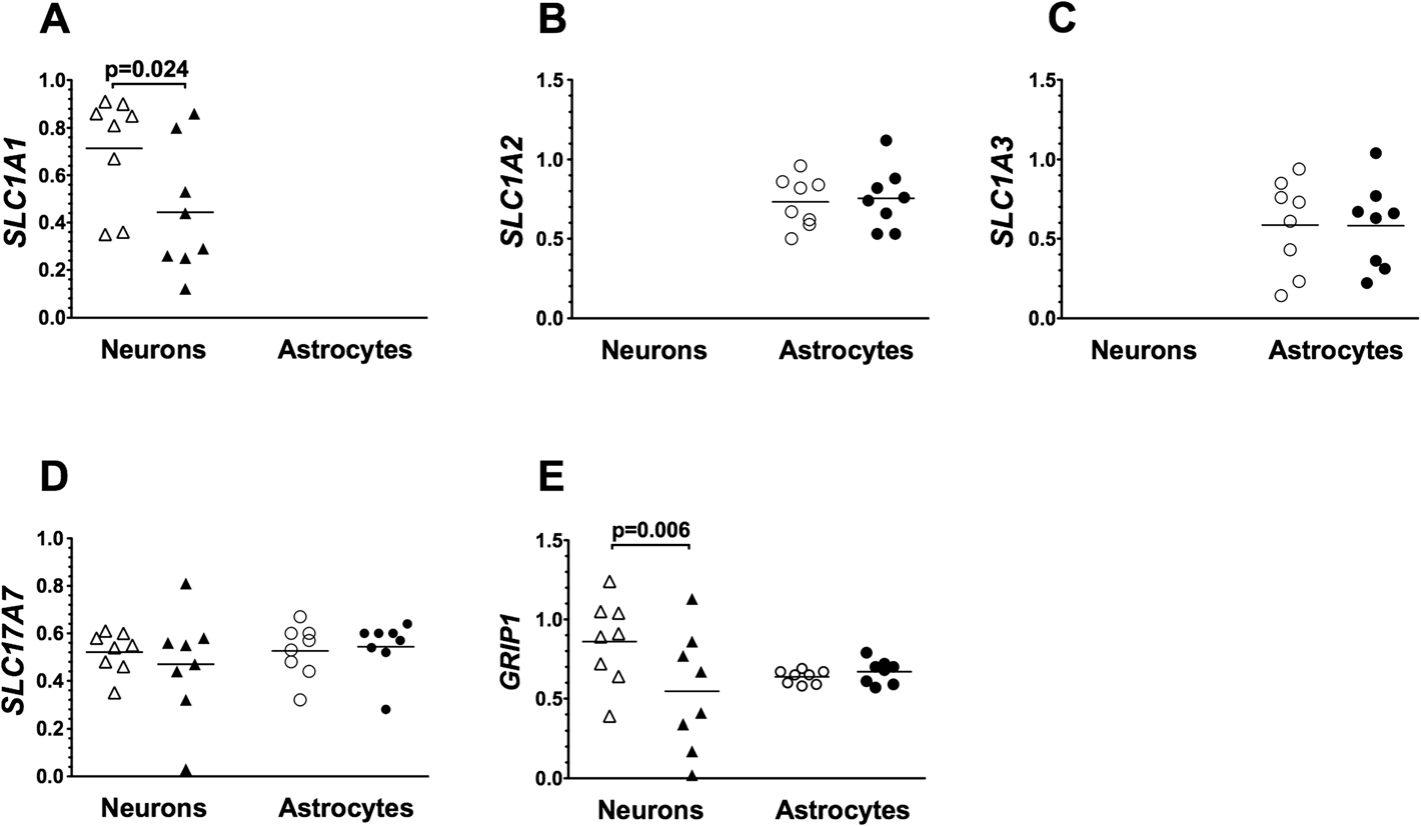
Levels of expression of glutamate transporter genes and a glutamate receptor-interacting gene **(A-E)**. Gene expression was measured in laser captured BA24 pyramidal neurons and separately in surrounding astrocytes of typically developing control donors (open symbols) and ASD donors (closed symbols). Gene expression levels are normalized to the averaged levels of expression of references genes (*GAPDH* and *RNA18S1*). Mean values are noted by horizontal lines, and statistical significance (uncorrected for the number of comparisons) is noted above the data points. See Table 2 for statistical results following correction for the number of comparisons.

Additional genes associated with glutamatergic neurotransmission were interrogated in BA24 pyramidal neurons and astrocytes, including glutamate transporter genes, *SLC1A1*, *SLC1A2*, *SLC1A3*, *SLC17A7*, and the glutamate receptor interacting gene, *GRIP1*. Of these genes, the expression of astrocyte-associated transporter genes *SLC1A2* and *SLC1A3* was not measured in neurons and the neuronal glutamate transporter gene *SLC1A1* was not measured in astrocytes. Levels of *SLC1A1* (*t* = 2.88; *P* = 0.024) and *GRIP1* (*t* = 3.84; *P* =0.006) gene expression were lower in pyramidal neurons captured from ASD donors as compared to matched control donors (Figure 3). However, statistical significance was lost when *P* values were corrected for the number of matched pair comparisons (Table 2). No significant differences in the levels of expression of any of the glutamate-related genes, *SLC1A2*, *SLC17A7*, and *GRIP1*, were observed in astrocytes comparing ASD to matched control donors (Figure 3).

### BDNF/NTRK2

**Figure 4:**
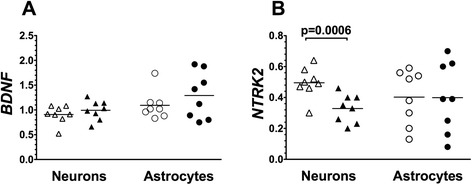
Levels of expression of *BDNF* **(A)** and its receptor gene *NTRK2* **(B)**. Gene expression was measured in laser captured BA24 pyramidal neurons and separately in surrounding astrocytes of typically developing control donors (open symbols) and ASD donors (closed symbols). Gene expression levels are normalized to the averaged levels of expression of references genes (*GAPDH* and *RNA18S1*). Mean values are noted by horizontal lines, and statistical significance (uncorrected for the number of comparisons) is noted above the data points. See Table 2 for statistical results following correction for the number of comparisons. *BDNF*, brain-derived neurotrophic factor.

Levels of expression for the neurotrophic factor gene *BDNF* and its receptor gene *NTRK2* were measured in pyramidal neurons and astrocytes from BA24 (Figure 4). *BDNF* expression in both neurons and astrocytes was similar comparing ASD to matched control donors. However, *NTRK2* expression levels were robustly lower in pyramidal neurons (*t* = 5.87; *P* = 0.0006), but not astrocytes, from ASD donors as compared to control donors. The difference in *NTRK2* expression levels between ASD and control donors remained statistically significant after correction for the number of matched comparisons (Table 2).

### Expression of selected genes in BA10

**Figure 5:**
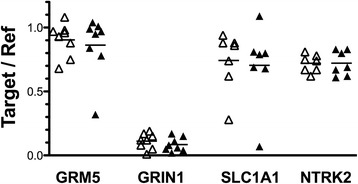
Levels of expression of *GRM5*, *GRIN1*, *SLC1A1*, and *NTRK2* in pyramidal neurons of BA10. Gene expression was measured in laser captured BA10 pyramidal neurons from typically developing control donors (open symbols) and ASD donors (closed symbols). Gene expression levels are normalized to the averaged levels of expression of references genes (*GAPDH* and *RNA18S1*). Mean values are noted by horizontal lines. No statistically significant differences were observed.

The expression levels of five genes were studied in BA10 pyramidal neurons from ASD and control donors. Chosen for study were those genes analyzed in BA24 pyramidal neurons that demonstrated either no difference (*GRM5*), marginal or modest differences (*GRIN1*, *SLC1A1*), or highly significant differences (*NTRK2*) comparing ASD to control donors as noted above. In this set of experiments, BA10 tissue from one age-matched pairs of donors used in the BA24 studies above was not available; BA10 tissue from one different age-matched pair of donors was substituted as noted in Table 1. The expression levels of each of these five genes in laser captured pyramidal neurons from BA10 were similar in ASD and matched controls (Figure 5; *GRIN1 t* = 0.74, *P* = 0.49; *GRM5 t* = 0.90, *P* = 0.40; *SLC1A1 t* = 0.26, *P* = 0.80; *NTRK2 t* = 0.03, *P* = 0.97).

### Demographic variables, reference genes, and tissue factors

There were no statistical differences between ASD and matched control donors of BA24 tissue regarding age, RIN or PMI values (Table 1). Likewise, there were no differences in these variables comparing the groups of donors of BA10 tissue (Table 1). There were no significant correlations between age or PMI and any of the 15 gene expressions measured in BA24 pyramidal neurons (Additional file 2). There was a significant correlation between levels of *GRIN2C* expression and RIN values, but there were no other correlations between RIN and the levels of any mRNA that was studied in BA24 pyramidal neurons. In BA24 astrocytes, age correlated significantly with *NTRK2* gene expression levels (*P* = 0.002) but not with levels of any other gene (Additional file 3). RIN did not significantly correlate with expression levels of any gene in BA24 astrocytes, while PMI correlated with only *SLC17A7* gene expression in BA24 astrocytes (Additional file 3). In BA10 pyramidal neurons, there were no significant correlations between age, RIN, or PMI and the levels of expression of any of the target or reference genes (Additional file 4).

Reference genes were carefully chosen as those that were stable in their expression levels across the two groups of donors. The ratios of levels of expression of *RNA18S1* and *GAPDH* were not significantly different in laser-captured BA24 neurons (*t* = 1.00, *P* = 0.35), BA24 astrocytes (*t* = 0.93, *P* = 0.38), or BA10 neurons (*t* = 1.82, *P* = 0.11) comparing ASD and matched control donors (see Additional file 5).

**Figure 6:**
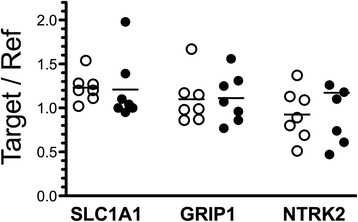
Ratio of reference gene expression levels. Gene expression was measured for *GAPDH* and *RNA18S1* in typically developing control donors (open symbols) and ASD donors (closed symbols). The ratio of gene expression between the two reference genes was compared for control and ASD subjects for BA24 neurons and astrocytes and BA10 neurons to ensure that the reference genes had stable expression levels between the groups. No statistically significant differences were observed.

It should be noted that we wished to screen multiple brain regions for these gene expression changes to determine the extent to which they occurred in the brain. Because LCM is time-intensive and expensive, we attempted to measure *SLC1A1*, *GRIP1*, and *NTRK2* expression in RNA isolated from homogenates of ACC. However, we were unable to detect any differences in expression of these genes in RNA isolates from homogenates of ACC, using tissues collected from seven of the eight ASD - control donor pairs that were used for the LCM study above (Figure 6). No significant difference was found for the ratios of expression of the reference genes *GAPDH* and *TATA* from the homogenate punch samples when comparing ASD and control donors (Additional file 6; *t* = 0.19, *P* = 0.86).

Four of the ASD donors had been prescribed psychotherapeutic medications, with three of the four receiving selective serotonin retake inhibitors (SSRI) antidepressants and four of the four receiving antipsychotic drugs (see Table 1). Considering that effects of psychotherapeutic drugs might contribute to observed differences in gene expression in BA24 neurons as shown above, a comparison was made of antidepressant/antipsychotic-exposed ASD donors to ASD donors not exposed to these drugs. Albeit the sample sizes of these two groups are small and that these two subgroups were not matched for age and other demographic variables, there were no significant difference in levels of *GRM8* (unpaired *t*-test; *t* = 0.17, *P* = 0.87), *GRIP1* (0.01, *P* = 0.99), *SLC1A1* (*t* = 0.28, *P* = 0.79), and *NTRK2* (*t* = 0.59, *P* = 0.57) comparing antidepressant/antipsychotic-exposed ASD donors to ASD donors without these drugs.

### Statistical considerations

Data from the matched control and ASD cases were analyzed using a paired *t*-test. Paired analyses were chosen because ASD and control donors were matched prior to the initiation of the analyses of gene expression levels based on several factors including, gender, age, and RNA quality values. These factors were considered extremely important for matching since each would be expected to impact measurements of gene expression levels. In addition, as noted in Methods, tissues from matched pairs of donors were processed on the same day so that RNA storage times were matched. Later, reverse transcription of RNA was performed on the same day for matched donor pairs, and PCR amplifications were performed simultaneously and run on the same plate. Given the meticulous care in the pairing of tissue donors and experiments that were performed on these tissues, we feel most confident with the results of the paired analyses of the resulting data. Alternative unpaired analyses could be used with the argument that all samples are taken from two separate groups of unrelated donors. To consider this, we subjected gene expression data from BA24 pyramidal neurons to MANOVA. The results of the MANOVA demonstrated a significant group effect (*F* = 563.3_(7,1)_; *P* = 0.03), with significantly lower expression levels of *NTRK2* (*P* = 0.002) in the ASD group as compared to the control group, similar to that observed in the paired analysis above. In addition, expression levels of *GRIN1* (*P* = 0.009) and *GRM8* (*P* = 0.02) were lower in ASD as compared to control donors.

## Discussion

This study examined the expression of genes involved in glutamatergic neurotransmission in ASD. We found a robust reduction in expression of a neurotrophin receptor gene (*NTRK2*) in pyramidal neurons dissected by LCM from ACC tissue of donors with ASD when compared to typically developing controls. In addition, there were trends towards low levels of expression of genes encoding a metabotropic glutamate receptor (*GRM8*), an ionotropic glutamate receptor subunit (*GRIN1*), a glutamate transporter (*SLC1A1*), and a glutamate receptor anchoring protein (*GRIP1*). This is the first study to use LCM to isolate a specific cell population to examine the molecular pathology of ASD. The use of LCM to capture distinct cell populations provided cellular resolution to the assessment of pathology and permitted identification of gene expression changes that we were unable to detect using homogenates of the same brain region in the same subjects. The difference in the results of LCM of single cell populations versus homogenates of tissues is likely the result of overlapping gene expression in other unaffected cell types that in essence dilute cell-specific gene expression changes, as has been demonstrated in research on Alzheimer’s disease [43,44]. The gene expression deficits identified in the present study were restricted to ACC pyramidal neurons and not found in surrounding astrocytes in the same brain region, nor in pyramidal neurons in BA10 of the prefrontal cortex, suggesting the presence of a cell- and region-specific pathology in ASD.

A striking finding of this study was the decreased expression of the growth factor receptor gene *NTRK2* in ACC pyramidal neurons from ASD donors. This gene encodes the high-affinity tyrosine kinase B receptor, TrkB, the primary ligand for which is BDNF, but neurotrophin-3 (NT-3) and neurotrophin-4/5 (NT-4/5) also can signal through the TrkB receptor [45,46]. TrkB signaling is critical to cell function, with ligand-activated TrkB engaging several intracellular signaling pathways including mitogen-activated protein kinase (MAPK), phosphoinositide 3-kinase (PI3), and extracellular-signal-regulated kinase (ERK) that are important in neurotransmission, plasticity, and differentiation [47]. Other evidence of abnormal BDNF/TrkB signaling in ASD includes several reports of elevated peripheral and central BDNF levels in ASD [34,48-51]. Given the multitude of effectors of TrkB receptor signaling, reduced TrkB signaling in ASD could have severe consequences on brain development and function. *NTRK2*-deficient mice die soon after birth [52,53], but mice with a conditional loss of *NTRK2* expression exhibit a wide variation in behavior ranging from social deficits to antisocial behavior (as reviewed by Lindholm and Castrén, 2014 [54]). It is noteworthy that the BTBR T^+^*tf*/J mouse model for ASD exhibits reduced TrkB protein expression [55]. Defective TrkB signaling has been shown in Angelman [56] and Fragile X [57] mouse models, both of which are syndromes with a high incidence of autistic features. The present study provides further support for a role of deficient BDNF/TrkB signaling in ASD, specifically implicating reduced TrkB signaling in ACC pyramidal neurons.

A genetic association between polymorphisms of *NTRK2* and ASD has been identified [34]. The most strongly associated polymorphisms in ASD were found in the intron-spanning regions of *NTRK2* [30]. It seems unlikely that a polymorphism in *NTRK2* would account for the reduction in *NTRK2* expression in pyramidal cells observed in the present study since one would expect the polymorphic *NTRK2* to similarly affect *NTRK2* expression in astrocytes and pyramidal neurons in other brain regions. Of the cells studied, we found the reduction of *NTRK2* expression only in ACC pyramidal neurons.

Cells used for this study were dissected from neocortical layer III of the ACC where both changes in cell size and packing density have been demonstrated previously [21]. An elevated number of neurons detected in layer VI of the ASD ACC have been interpreted as a possible incomplete migration of neurons during development [21]. Mini-column disturbances, that is, reduced column width and increased column number in ASD, have been shown in the ACC with pyramidal neurons demonstrating misalignment [58]. Stoner *et al*. [59] recently reported abnormal columnar architecture in several cortical regions involving all six cortical layers, based on abnormal gene expressions and areas referred to as “patches” [59] with low gene expression levels. The ACC was not investigated in this later study. In addition, an increase in spine density of pyramidal neurons in frontal, parietal, and temporal lobes has been reported for ASD [60], although spine density specifically in the ACC has not been studied to date. Interestingly, BDNF/TrkB signaling plays a prominent role in synaptogenesis and synaptic remodeling of excitatory neurons during development [61,62]. Hence, low levels of *NTRK2* expression in glutamatergic pyramidal neurons in ASD observed in the present study may be etiologically linked to abnormalities in cortical columnar architecture in ASD previously reported.

Abnormalities in neurotransmitter signaling networks are likely to be a core component of the cellular neurobiology of ASD. Gene expression changes in synapse-related genes have been demonstrated in disorders that have autistic features such as Angelman syndrome and Fragile X syndrome (as reviewed by Ebert and Greenberg, 2013 [63]). We chose to interrogate potential expression changes in several genes associated with excitatory amino acid cell signaling in autism. Four of these genes, *SLC1A1*, *GRIP1*, *GRIN1*, and *GRM8*, demonstrated trends towards reduced expression in ACC pyramidal neurons in ASD. SLC1A1 is responsible for glutamate uptake from the synapse by pyramidal neurons while GRIP1 anchors AMPA receptors to the cell membrane. GRIN1 is the mandatory subunit of the N-methyl-D-aspartate (NMDA) receptor and GRM8 inhibits the adenylyl cyclase/cAMP pathway and decreases the likelihood of cell death associated with excess NMDA signaling [64]. The decreased expression of *GRM8* receptor in ASD, if accompanied by reduced GRM8 receptor, could leave pyramidal cells susceptible to neurotoxic effects of elevated glutamate signaling through the NMDA receptor. This neurotoxic effect could be amplified further if reduced *SLC1A1* expression observed in ASD results in less glutamate transporter activity at the glutamatergic synapse, thereby further increasing glutamate activation of NMDA receptors on these pyramidal neurons. Reduced *GRIP1* expression, as we observed in ASD, could be a compensatory mechanism to override excess NMDA receptor activation by glutamate. Whatever the exact sequence or consequence, abnormal expression levels of these genes imply that excitatory output from the ACC via pyramidal neurons is disrupted in ASD. Because multiple cell types express GRIP1 and NTRK2, it cannot be dismissed that these glutamatergic-related gene expression decreases are only found in excitatory cells. While we did not find *GRIP1* or excitatory transporter changes (*SLC1A2* and *SLC1A3*) in the glia cells studied herein, altered expression of these genes could occur in inhibitory neurons of the ACC, which were not studied. Nevertheless, any disruption of glutamate transmission and function implicates a compromise in the delicate balance of inhibitory and excitatory neuronal activity of the ACC. Future studies need to include a more detailed examination of gene expression in other cell types of the ACC.

### Limitations

There were several limitations in the current study that should be taken into consideration. First, like many studies of ASD relying on postmortem brain tissue, the number of donors available was limited and reduced the power of the study. It should be noted that all donors that had a history of seizure disorder were excluded from the study to reduce experimental variability. It is worth noting that our laboratory has observed that the study of gene expression in distinct cell populations captured by LCM results in reduced variability of data, permitting the use of smaller sample sizes to obtain statistical significance. ASD is a highly heterogeneous disorder, and there were not enough brain donors to permit us to examine the gene expressions in subgroups of donors based on clinical presentation. Changes in gene expression do not always correlate with changes in the expression of the cognate protein, and often a change in gene expression is not temporally correlated with changes in the cognate proteins. Hence, whether reduced *NTRK2* expression in ASD pyramidal neurons results in lower TrkB protein in these cells is not currently known. Nevertheless, altered levels of *NTRK2* expression in ASD is highly suggestive of perturbed TrkB signaling in ASD. ASD donors used in the present study were exposed to psychotherapeutic drugs that could alter gene expression, particularly SSRI and risperidol, an atypical antipsychotic. But, repeated administration of the antidepressants tranylcypromine, sertraline, or desipramine to rats increases *NTRK2* expression [65]. Hence, reduced *NTRK2* expression in the present study is unlikely to reflect previous antidepressant drug therapy. Repeated treatment of rats with risperidol produced no change in TrkB immunoreactivity in the brain [66], again suggesting that psychotherapeutic drug exposure is not a likely mediator of reduced *NTRK2* expression in ASD in the present study. Findings herein demonstrate no obvious effect of psychotherapeutic drug exposure on *NTRK2* (or *GRIN1*, *GRM8*, *GRIP1*, *SLC1A1*) expression. Finally, tissue-related physical factors were considered as possible sources of variance in these experiments. However, there were no significant differences between ASD and control donor samples when comparing age, RNA quality (RIN), and postmortem intervals (Table 1).

## Conclusions

Multiple gene abnormalities are significantly associated with ASD [67]. It therefore seems likely that multiple etiologies exist that can result in ASD. Brain pathology is downstream to gene polymorphisms and etiologies and more proximal to the pathophysiology underlying the abnormal behaviors. Hence, the neuropathology of ASD may be more limited in terms of variability than the genotypes that can contribute to it, that is, different etiologies may result in a similar structural/neurochemical abnormalities that disrupt function to produce a specific set of behavioral abnormalities. The present study was designed to examine possible disruption of a key neuronal unit, the glutamatergic pyramidal neuron, of the ACC in ASD. The data presented here reveals strong evidence of disrupted neurochemistry of ACC pyramidal neurons, specifically involving glutamatergic neurotransmission and neurotrophic factor (BDNF) signaling through the TrkB receptor. It is tempting to speculate that abnormalities in ACC as revealed in imaging studies of ASD patients [4-7,9,11-16,68] and pathology research using postmortem brain tissues [21-25] are at least partially the result of deficits in pyramidal neuronal function in the ACC as revealed in the present study. Pyramidal neurons in layer III of the ACC synapse with other cortical neurons including inhibitory neurons and long-range pyramidal motor neurons in layer V and the present findings suggest that neural communication with these other brain areas or pathways is altered in ASD. Interestingly, pyramidal neurons take years to reach maturity, possibly well into adolescence [69], indicating that normal neural development of this cell type may be especially vulnerable to deleterious environmental influences occurring early in life.

## Additional files

Additional file 1:
**Primer information.**
Additional file 2:
**Pearson’s correlation analyses for possible relationships between gene expression levels in BA24 neurons and age, RNA quality (RIN), and postmortem interval (PMI).**
Additional file 3:
**Pearson’s correlation analyses for possible relationships between gene expression levels in BA24 astrocytes and age, RNA quality (RIN), and postmortem interval (PMI).**
Additional file 4:
**Pearson’s correlation analyses for possible relationships between gene expression levels in BA10 neurons and age, RNA quality (RIN), and postmortem interval (PMI).**
Additional file 5:
**Ratio of reference gene expression levels.** Gene expression was measured for *GAPDH* and *RNA18S1* in typically developing control donors (open symbols) and ASD donors (closed symbols). The ratio of gene expression between the two reference genes was compared for control and ASD subjects for BA24 neurons and astrocytes and BA10 neurons to ensure that the reference genes had stable expression levels between the groups. No statistically significant differences were observed. Additional file 6:
**Ratio of reference gene expression levels for qPCR.** Gene expression was measured for *GAPDH* and *TATA* in typically developing control donors (open symbols) and ASD donors (closed symbols). The ratio of gene expression between the two reference genes were compared for control and ASD subjects for BA24 punch-dissected gray matter tissue samples to ensure that the reference genes had stable expression levels between the groups. No statistically significant difference was observed.

## Abbreviations

ACC: anterior cingulate cortex
ASD: autism spectrum disorder
BA10: Brodmann area 10
BA24: Brodmann area 24
Ct: cycle threshold
EEG: electroencephalogram
LCM: laser capture microdissection
MANOVA: multivariate analysis of variance
PET: positron emission tomography
PMI: postmortem interval
qPCR: real-time quantitative polymerase chain reaction
RIN: RNA integrity number
SPECT: single-photon emission computerized tomography
SSRI: selective serotonin retake inhibitors
