# Erratum to: *NTRK2* expression levels are reduced in laser captured pyramidal neurons from the anterior cingulate cortex in males with autism spectrum disorder

**DOI:** 10.1186/s13229-015-0033-0

**Published:** 2015-06-20

**Authors:** Michelle J Chandley, Jessica D Crawford, Attila Szebeni, Katalin Szebeni, Gregory A Ordway

**Affiliations:** Department of Health Sciences, College of Public Health, East Tennessee State University, P.O. Box 70673, Johnson City, TN 37614 USA; Department of Biomedical Sciences, James H. Quillen College of Medicine, East Tennessee State University, P.O. Box 70582, Johnson City, TN 37614 USA

## Erratum

Upon publication of [1], it was noticed by the author that the caption for figure six was incorrect. The correction caption for figure six is as follows:

Figure six. Levels of expression of SLC1A1, GRIP1, and NTRK2 in punch-dissected BA24 gray matter. Gene expression was measured in typically developing control donors (open symbols) and ASD donors (closed symbols) using RNA isolated from homogenates of punched-dissected gray matter rather than specific laser captured cells as shown in Figs. 1-5. Gene expression levels were determined by real time PCR and are normalized to the geometric mean of stable references genes (GAPDH, TBP, and Ribo18S1). No statistically significant differences were observed.

## References

[1] Chandley MJ, Crawford JD, Szebeni A, Szebeni K, Ordway GA (2015). *NTRK2* expression levels are reduced in laser captured pyramidal neurons from the anterior cingulate cortex in males with autism spectrum disorder. Mol Autism.

