# Supplementary material for: NTRK2 expression levels are reduced in laser captured pyramidal neurons from the anterior cingulate cortex in males with autism spectrum disorder
Source: Mol Autism. 2015 May 16;6:28. doi: 10.1186/s13229-015-0023-2 (PMC4440594; doi:10.1186/s13229-015-0023-2)
Supplement: Additional file 1: — Primer information. [file 13229_2015_23_MOESM1_ESM.pdf]

Additional File 1. Primer information

Target or Reference	Genbank Accession Number	Primer Sequence	PCR Product Size (bp)
AQP4	NM_001650<br>NM_004028	(f) TCCAAACGGACTGATGTCACTGGCT<br>(r) CAAAGGATCGGGCGGGATTCATGC	118
BDNF	NM_170735<br>NM_170732<br>NM_170731<br>NM_001709<br>NM_170733<br>NM_170734	(f) AGAGCCCTGTATCAACCCAGAAACACC<br>(r) GCAATGCCAACTCCACATAGCCTCC	112
GAPDH	NM_002046	(f) TGCACCACCAACTGCTTAGC<br>(r) GGCATGGACTGTGGTCATGAG	87
GRIA1	NM_001200	(f) GTGCGCAGCTTCCACCATGAA<br>(r) CTGAGGTGATAAACTCCTCCGTGGG	122
GRIK2	NM_000830<br>NM_175611	(f) ATTGACTCCAAAGGTTACGGAGTGGG<br>(r) GCAGCTTCCCTTCTTCTTGGAGTTGA	100
GRIN1	NM_000832<br>NM_021569<br>NM_007327	(f) CCTGGAAGCAGAACGTCTCCCTGT<br>(r) TGCTGCGCGAGTCACATTCCTGAT	108
GRIN2A	NM_001134407<br>NM_000833<br>NM_001134408	(f) TCGACCTGGCCTTGCTTCAGTTTGT<br>(r) GCTGGCTGCTCATCACCTCGTTCTT	111
GRIN2B	NM_000834	(f) CCTCATCACCTTCATCTGCG<br>(r) CATGGATGCAGCTGTAGATACC	125
GRIN2C	NM_000835	(f) TGGTGGCCATCACCGTCTTCAT<br>(r) CCACACGGACTTGCCGATAGTGA	116
GRIN2D	NM_000836	(f) CTTCGTGGAGACCGGCATCAGCGTC<br>(r) ACGAACATCATCACCCACACGGCGG	108
GRIP1	NM_021150	(f) GCCACAGAAACTCTCTCTTCTCCACC	96

	NM_001178074	(r) CACTCTGTCTCCAATCTGTAGCACCC	
GRM5[a]	NM_000842	(f) GCCAGCAGATCCAGCAGCCTAGTCA<br>(r) TCATTCTGGGCCCACGTGACGGATT	101
GRM5[b]	NM_000842<br>NM_001143831	Not available, purchased from Qiagen	125
GRM8	NM_000845<br>NM_001127323<br>NM_001127326<br>NR_028041	Not available, purchased from Qiagen	78
MOG	NM_002433.3<br>NM_206809.2	(f) CCTGCTGGAAGATAACCCTGTTTG<br>(r) CACTCAGAAGGGATTTCGTAGCTC	134
NTRK2	NM_006180<br>NM_001007097<br>NM_001018064<br>NM_001018065<br>NM_001018066	(f) TGTAGTGTGGCAGGTGATCCGGT<br>(r) GGAGCCCTGTGTGTGGCTTGTTT	96
RNA18S1	NR_003286	(f) GTAACCCGTTGAACCCCATT<br>(r) CCATCCAATCGGTAGTAGCG	131
SLC1A1[a]	NM_004170	Not available, purchased from Qiagen	110
SLC1A1[b]	NM_004170	(f) CCTGAAGTCAGTACGGTGGATGCC<br>(r) GGGAGGCTTCACTTCTTCACGCTT	117
SLC1A2	NM_004171	(f) CAAGCTTGGATCACTGCCCTGG<br>(r) GGTTGCTCCAACAGGAAGGACGA	129
SLC1A3	NM_004172	(f) TGCAAGCACTCATCACCGCTCTGGG<br>(r) ACGCGCTTGTCCACGCCATTGTTCT	100
TATA	NM_003194	(f) CACTTCGTGCCCGAAACGCCGAAT<br>(r) ATCAGTGCCGTGGTTCGTGGCTCT	88
UBC	NM_021009	(f) ATTTGGGTCGCGGTTCTTG<br>(r) TGCCTTGACATTCTCGATGGT	133
VGlut1	NM_020309	(f) TCGGAGAGAGCGCGAAACTCAT<br>(r) TGGCCACGATGATGGCATAGACT	99

[a] Denotes use in RT-PCR experiments
[b] Denotes use in Endpoint-PCR experiments
