# Supplementary material for: NTRK2 expression levels are reduced in laser captured pyramidal neurons from the anterior cingulate cortex in males with autism spectrum disorder
Source: Mol Autism. 2015 May 16;6:28. doi: 10.1186/s13229-015-0023-2 (PMC4440594; doi:10.1186/s13229-015-0023-2)
Supplement: Additional file 2: — Pearson’s correlation analyses for possible relationships between gene expression levels in BA24 neurons and age, RNA quality (RIN), and postmortem interval (PMI). [file 13229_2015_23_MOESM2_ESM.pdf]

Additional File 2. Pearson's correlation analyses for possible relationships between gene expression levels in BA24 neurons and age, RNA quality (RIN) and postmortem interval (PMI).

Gene[a] (protein[b])	Statistic	Age	RIN	PMI
RNA18S1 / GAPDH[c]	Pearson Correlation<br>Sig. (2-tailed)<br>N	-.309<br>.243<br>16	.043<br>.875<br>16	-.250<br>.350<br>16
GRIN1 (NR1)	Pearson Correlation<br>Sig. (2-tailed)<br>N	.050<br>.855<br>16	.318<br>.231<br>16	-.226<br>.400<br>16
GRIN2A (NR2A)	Pearson Correlation<br>Sig. (2-tailed)<br>N	-.055<br>.851<br>14	.531<br>.051<br>14	.176<br>.548<br>14
GRIN2B (NR2B)	Pearson Correlation<br>Sig. (2-tailed)<br>N	.109<br>.687<br>16	.579<br>.019<br>16	-.250<br>.351<br>16
GRIN2C (NR2C)	Pearson Correlation<br>Sig. (2-tailed)<br>N	.229<br>.394<br>16	.642[d]<br>.007<br>16	-.091<br>.737<br>16
GRIN2D (NR2D)	Pearson Correlation<br>Sig. (2-tailed)<br>N	.322<br>.224<br>16	.082<br>.763<br>16	.203<br>.452<br>16
GRIA1 (GluR-1)	Pearson Correlation<br>Sig. (2-tailed)<br>N	-.374<br>.154<br>16	.213<br>.429<br>16	-.012<br>.966<br>16
GRIK2 (GRIK2, GluK2)	Pearson Correlation<br>Sig. (2-tailed)<br>N	-.316<br>.233<br>16	.250<br>.350<br>16	-.346<br>.189<br>16
GRM5 (mGluR5)	Pearson Correlation<br>Sig. (2-tailed)<br>N	-.017<br>.953<br>15	-.117<br>.677<br>15	-.435<br>.105<br>15
GRM8 (mGluR8)	Pearson Correlation<br>Sig. (2-tailed)<br>N	.131<br>.684<br>12	.140<br>.664<br>12	.029<br>.929<br>12
SLC1A1 (EAAT3)	Pearson Correlation<br>Sig. (2-tailed)	-.191<br>.478	.285<br>.284	-.591<br>.016

	N	16	16	16
SLC17A7 (VGlut1)	Pearson Correlation	.003	.312	-.358
	Sig. (2-tailed)	.991	.240	.173
	N	16	16	16
GRIP1 (GRIP1)	Pearson Correlation	-.128	.265	-.030
	Sig. (2-tailed)	.638	.320	.913
	N	16	16	16
BDNF (BDNF)	Pearson Correlation	.005	-.084	-.212
	Sig. (2-tailed)	.985	.757	.430
	N	16	16	16
NTRK2 (NTRK2,TrkB)	Pearson Correlation	.522	.271	-.076
	Sig. (2-tailed)	.038	.310	.780
	N	16	16	16

[a] Derived from the HUGO gene nomenclature committee responsible for designating unique gene names (Wain et al., 2002).

[b] Official protein nomenclature showing both the recommended and alternative protein names (Uniprot, 2008).

[c] Ratio of the two reference genes; all other expression data are normalized to the average of these two reference genes.

[d] Correlation is significant at $p<0.01$ (2-tailed).
