# Supplementary material for: NTRK2 expression levels are reduced in laser captured pyramidal neurons from the anterior cingulate cortex in males with autism spectrum disorder
Source: Mol Autism. 2015 May 16;6:28. doi: 10.1186/s13229-015-0023-2 (PMC4440594; doi:10.1186/s13229-015-0023-2)
Supplement: Additional file 3: — Pearson’s correlation analyses for possible relationships between gene expression levels in BA24 astrocytes and age, RNA quality (RIN), and postmortem interval (PMI). [file 13229_2015_23_MOESM3_ESM.pdf]

**Additional File 3.** Pearson's correlation analyses for possible relationships between gene expression levels in BA24 astrocytes and age, RNA quality (RIN) and postmortem interval (PMI).

| Gene[a] (protein[b]) | Statistic | Age | RIN | PMI |
|---|---|---|---|---|
| *RNA18S* / *GAPDH*[c] | Pearson Correlation | .074 | .119 | .443 |
| | Sig. (2-tailed) | .786 | .662 | .085 |
| | N | 16 | 16 | 16 |
| *GRIN1* (NR1) | Pearson Correlation | .092 | .133 | -.003 |
| | Sig. (2-tailed) | .736 | .624 | .991 |
| | N | 16 | 16 | 16 |
| *GRIN2A* (NR2A) | Pearson Correlation | .282 | .516 | -.116 |
| | Sig. (2-tailed) | .290 | .041 | .670 |
| | N | 16 | 16 | 16 |
| *GRIN2B* (NR2B) | Pearson Correlation | .523 | .520 | -.060 |
| | Sig. (2-tailed) | .038 | .039 | .826 |
| | N | 16 | 16 | 16 |
| *GRIN2D* (NR2D) | Pearson Correlation | .547 | .395 | .227 |
| | Sig. (2-tailed) | .028 | .130 | .398 |
| | N | 16 | 16 | 16 |
| *GRIA1* (GluR-1) | Pearson Correlation | -.046 | .490 | .009 |
| | Sig. (2-tailed) | .865 | .054 | .975 |
| | N | 16 | 16 | 16 |
| *GRM5* (mGluR5) | Pearson Correlation | .577 | .328 | .112 |
| | Sig. (2-tailed) | .019 | .216 | .679 |
| | N | 16 | 16 | 16 |
| *SLC1A3* (EAAT1) | Pearson Correlation | -.340 | -.238 | -.344 |
| | Sig. (2-tailed) | .197 | .375 | .192 |
| | N | 16 | 16 | 16 |
| *SLC1A2* (EAAT2) | Pearson Correlation | -.101 | .023 | -.186 |
| | Sig. (2-tailed) | .711 | .932 | .491 |
| | N | 16 | 16 | 16 |
| *SLC17A7* (vGlut1) | Pearson Correlation | -.470 | .214 | -.655[d] |
| | Sig. (2-tailed) | .066 | .426 | .006 |
| | N | 16 | 16 | 16 |
| *GRIP1* (GRIP1) | Pearson Correlation | -.086 | -.146 | .378 |
| | Sig. (2-tailed) | .751 | .590 | .149 |
| | N | 16 | 16 | 16 |
| *BDNF* (BDNF) | Pearson Correlation | .479 | -.153 | .492 |
| | Sig. (2-tailed) | .060 | .571 | .053 |
| | N | 16 | 16 | 16 |
| *NTRK2* (NTRK2, TrkB) | Pearson Correlation | .711[d] | .123 | .187 |
| | Sig. (2-tailed) | .002 | .649 | .488 |
| | N | 16 | 16 | 16 |

[a] Derived from the HUGO gene nomenclature committee responsible for designating unique gene names (Wain et al., 2002).

[b] Official protein nomenclature showing both the recommended and alternative protein names (Uniprot, 2008).

[c] Ratio of the two reference genes; all other expression data are normalized to the average of these two reference genes.

[d] Correlation is significant at $p<0.01$ (2-tailed).
