# Supplementary material for: NTRK2 expression levels are reduced in laser captured pyramidal neurons from the anterior cingulate cortex in males with autism spectrum disorder
Source: Mol Autism. 2015 May 16;6:28. doi: 10.1186/s13229-015-0023-2 (PMC4440594; doi:10.1186/s13229-015-0023-2)
Supplement: Additional file 4: — Pearson’s correlation analyses for possible relationships between gene expression levels in BA10 neurons and age, RNA quality (RIN), and postmortem interval (PMI). [file 13229_2015_23_MOESM4_ESM.pdf]

Additional File 4. Pearson's correlation analyses for possible relationships between gene expression levels in BA10 neurons and age, RNA quality (RIN) and postmortem interval (PMI).

**Gene[a] (protein[b])**	**Statistic**	**Age**	**RIN**	**PMI**
RNA18S1 / GAPDH[c]	Pearson Correlation	-.122	-.407	.002
	Sig. (2-tailed)	.653	.148	.995
	N	16	14	14
GRIN1 (NR2A)	Pearson Correlation	.114	.415	.059
	Sig. (2-tailed)	.675	.140	.841
	N	16	14	14
GRM5 (mGluR5)	Pearson Correlation	.328	.228	.088
	Sig. (2-tailed)	.215	.432	.765
	N	16	16	14
SLC1A1 (EAAT3)	Pearson Correlation	-.105	.659	.282
	Sig. (2-tailed)	.722	.020	.374
	N	14	12	12
NTRK2 (NTRK2, TrkB)	Pearson Correlation	-.234	.537	-.094
	Sig. (2-tailed)	.420	.072	.773
	N	14	12	12

[a] Derived from the HUGO gene nomenclature committee responsible for designating unique gene names (Wain et al., 2002).

[b] Official protein nomenclature showing both the recommended and alternative protein names (Uniprot, 2008).

[c] Ratio of the two reference genes; all other expression data are normalized to the average of these two reference genes.
