# Supplementary material for: NTRK2 expression levels are reduced in laser captured pyramidal neurons from the anterior cingulate cortex in males with autism spectrum disorder
Source: Mol Autism. 2015 May 16;6:28. doi: 10.1186/s13229-015-0023-2 (PMC4440594; doi:10.1186/s13229-015-0023-2)
Supplement: Additional file 5: — Ratio of reference gene expression levels. Gene expression was measured for GAPDH and RNA18S1 in typically developing control donors (open symbols) and ASD donors (closed symbols). The ratio of gene expression between the two reference genes was compared for control and ASD subjects for BA24 neurons and astrocytes and BA10 neurons to ensure that the reference genes had stable expression levels between the groups. No statistically significant differences were observed. [file 13229_2015_23_MOESM5_ESM.pdf]

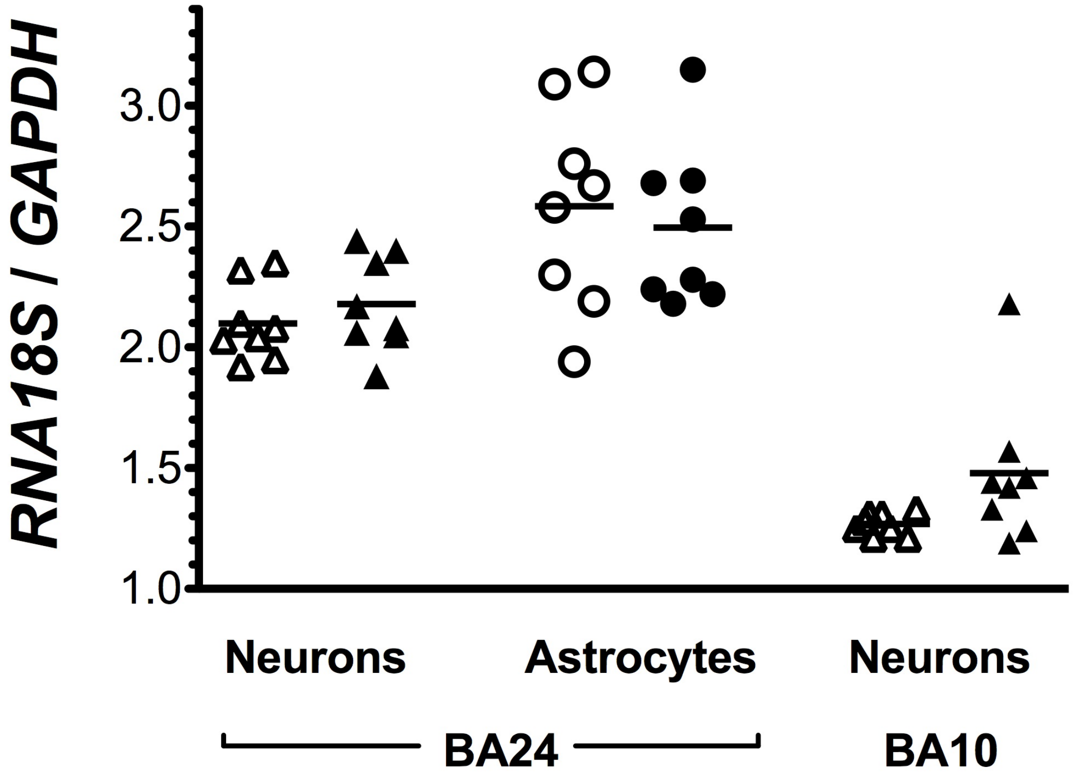

Additional File 5. Ratio of reference gene expression levels.

Gene expression was measured for *GAPDH* and *Ribo18S1* in typically developing control donors (open symbols) and ASD donors (closed symbols). The ratio of gene expression between the two reference genes were compared for control and ASD subjects for BA24 neurons and astrocytes and BA10 neurons to ensure that the reference genes had stable expression levels between the groups. No statistically significant differences were observed.
